# Peritoneal Reactivity Evaluation in Horses Subjected to Experimental Small Colon Enterotomy and Treated with Subcutaneous Heparin

**DOI:** 10.1155/2014/385392

**Published:** 2014-11-11

**Authors:** Juliana de Moura Alonso, Karoline Alves Rodrigues, Ana Lúcia Miluzzi Yamada, Marcos Jun Watanabe, Ana Liz Garcia Alves, Celso Antonio Rodrigues, Carlos Alberto Hussni

**Affiliations:** School of Veterinary Medicine and Animal Science, UNESP, University Estadual Paulista, P.O. Box 560, 18618-970 Botucatu, SP, Brazil

## Abstract

Heparin is routinely administered in postoperative abdominal surgery aiming to prevent adhesions formation; however, there is no consensus indicating its effectiveness. This study evaluated the effect of heparin on peritoneal reactivity after abdominal surgery, through the association between peritoneal fluid features and ultrasonographic and laparoscopic examination. Ten adult horses were used: control group (CG) and treated group (TG). Both groups underwent laparotomy and small colon enterotomy. TG received subcutaneous heparin at 150 IU/kg every 12 hours for 5 days. The animals underwent ultrasonography and peritoneal fluid examination prior to enterotomy (M0) 12 hours (M1), 1 day (M2), 2 days (M3), 4 days (M4), 6 days (M5), 10 days (M6), and 14 days after enterotomy (M7) with laparoscopic examination being performed on the fifth postoperative day. Peritoneal inflammatory response was observed in both groups. The peritoneal fluid of TG animals showed higher echogenicity during heparin therapy. No inflammatory difference was observed between groups through peritoneal fluid features, except for the higher D-dimer concentration in CG. On laparoscopy, slightly diffuse peritoneal reactivity for both groups was observed, being higher for TG. Laparoscopy and ultrasonography association allowed detailed access to the abdominal cavity. Ultrasonography assessed the diffuse peritoneal inflammation, and laparoscopy allowed the detailed analysis of the segments. No gross beneficial reactions resulting from the use of heparin on peritoneal reactivity were observed; however, it was observed by D-dimer evaluation that the TG had less fibrin deposition, which is directly related to a lower rate of abdominal adhesions formation.

## 1. Introduction

Enterotomies are often employed for enteroliths, foreign bodies, and impacted material removal [1, 2]. Surgical trauma results in peritoneal inflammation. The peritoneal response to an inflammatory stimuli of infectious nature or not is called peritonitis [3, 4].

After surgical trauma, peritoneal inflammation occurs concurrently with the coagulation process focusing on tissue reparative order. Thrombin is the major link between inflammation and coagulation system, since the enzyme is responsible for cleavage of circulating soluble fibrinogen to insoluble fibrin clot [5]. The coagulation process is counterbalanced by the fibrinolytic system, where the plasminogen activator (secreted by mesothelial cells, leukocytes, and tissues) acts cleaving plasminogen, the plasma protein that binds to fibrin clot to generate plasmin, a protease that acts in the lyses of fibrin [5–7].

If a persistent postsurgical inflammatory stimulus is present, an imbalance between the formation and lyses of fibrin may occur, giving rise to fibrinous adhesions which subsequently form the basis for fibrous abdominal adhesions development [8–10].

In this sense, the use of anticoagulants in order to prevent fibrin clots formation possibly inhibits adhesions formation [10, 11]. Heparin is an acid sulfated proteoglycan with molecular weight variation. A small fraction of heparin is responsible for the main anticoagulant effect. This fraction binds to antithrombin III, which is a slow thrombin, plasmin, and coagulation factors inhibitor. Heparin catalyzes the antithrombin III inhibition reaction [12], by stimulating tissue plasminogen activator (tPA) and therefore enhancing plasminogen activation, which results in fibrinolysis [12, 13].

Due to the large size of the horse abdomen, there is no method able to evaluate fully the abdominal cavity, being necessary the combination of different methods to better evaluation. It is described that the association of ultrasonography and laparoscopy allows greater accuracy in horses digestive system diseases evaluation [14, 15].

Abdominal ultrasonography is a noninvasive and widely available modality that can easily integrate the examination of the abdominal cavity [15, 16]. Laparoscopy has been expanding in abdominal disorders evaluation [15, 17, 18].

The physical chemical and cytological evaluation of peritoneal fluid [19] is seen as test of choice for peritoneal diseases investigation and presents easy implementation, security, and wealth of information [20].

Besides this, quantification of D-dimers can also be used as a tool for peritoneal response evaluation; D-dimer is a fragment that is released exclusively during fibrin lysis via the action of plasmin. In the serum, plasmin levels are correlated with the destruction of fibrin after hyperfibrinogenic and hypercoagulable states and serve as a marker of coagulation and fibrinolytic activity [21–23].

Few studies have been conducted using peritoneal fluid, and most of these have focused on human patients. However, measurement of the D-dimer concentration in the peritoneal fluid of equines with severe gastrointestinal disorders demonstrated marked hyperfibrinolysis related to increased fibrin formation and degradation [22]. A D-dimer concentration >4000 ng/mL has been established as the cut-off point for predicting poor prognosis in equines with abdominal afflictions [23].

This study aimed to evaluate heparin effect on peritoneal reactivity after laparotomy and experimental small colon enterotomy, associating peritoneal fluid features, laparoscopy, and ultrasonography examinations, since heparin is routinely used in the postoperative of abdominal surgery as a prophylactic strategy for adhesion formation inhibition, but without the existence of consistent results of its effectiveness [24].

## 2. Materials and Methods

Ten adult horses underwent a laparotomy and experimental small colon enterotomy in the standing position. Animals were divided into two groups of five animals: control group (CG) and treated group (TG). The TG immediately after surgery and every 12 hours for 5 days received heparin at a dose of 150 IU/kg subcutaneously. The CG did not receive treatment.

Prior to surgery, the right abdominal region was shaved and the horses of both groups received a combination of potassium penicillin (30.000 IU/kg intravenously), gentamicin^2^ (6.6 mg/kg intravenously), and flunixin meglumine (1.1 mg/kg intravenously). The procedure was performed in the standing position and under continuous infusion sedation with detomidine (5 *μ*g/kg bolus followed by continuous infusion of 20 *μ*g/kg/h) associated with butorphanol (20 *μ*g/kg followed by continuous infusion of 13 *μ*g/kg/h). After antisepsis, local anesthesia was performed on line incision and adjacent deep tissues with 2% lidocaine associated with vasoconstrictor.

A ten cm incision was performed on paralumbar fossa skin; subcutaneous tissue was divulsioned and muscle sectioned; the peritoneum was punctured and abdominal cavity accessed. Small colon identification was proceeded by direct palpation, and the further aboral segment was externalized; after delimitation and emptying of the enterotomy site segment, two coprostasis forceps were applied in order to prevent fecal flow. Subsequently two Allis clamps were applied for segment positioning and enterotomy realization in the band region; a simple separate suture pattern with 3–0 Catgut^7^ was applied. After suturing, the serosal layer was subjected to abrasion with dry gauze in 50 repeated moves for each adjacent portion of the band, which results in a hemorrhagic local serosa. The segment was repositioned and abdominal wall synthesis was performed in Sultan pattern with number 2 polyglactin 910; skin suture was performed in Wolff pattern with 0-monofilamentar nylon.

After surgery, benzathine penicillin was administered (30.000 IU/kg intramuscularly every 72 hours for 10 days), and gentamicin (6.6 mg/kg intravenously every 24 hours for 05 days), flunixin meglumine (1.1 mg/kg intramuscularly every 24 hours for 5 days), and antitetanus serum (10.000 UI subcutaneously in a single application) were administered for both groups.

The animals underwent ultrasonography and peritoneal fluid evaluation prior to enterotomy (M0); 12 hours (M1); 1 day (M2); 2 days (M3); 4 days (M4); 6 days (M5); 10 days (M6), and 14 days after enterotomy (M7).

The ultrasonography evaluation of small colon exam was performed with a convex transducer (5 MHz and 25–35% gain). To review the peritoneum and free peritoneal fluid a linear transducer (13 MHz and a gain of 80–90%) was used.

Ultrasonography examination was performed by transabdominal via and aimed to evaluate the small colon and peritoneal fluid. The small colon was visualized in the left dorsal quadrant, using as reference the left kidney, since the small colon is usually located caudal to it and dorsal to pelvic flexure. The small colon was evaluated for its topographical location, architecture, and wall thickening.

The parietal peritoneum was evaluated in the ventral portion of the abdomen. The presence of irregularities, fibrin deposition, and peritoneal thickening from possible peritoneal inflammation were evaluated. In the same region, the quantity, echogenicity, and presence of debris on free peritoneal fluid were assessed.

For ultrasonographic findings standardization a score scheme to quantify peritoneal fluid and evaluate its echogenicity was created.

Because the dynamic feature of ultrasonography examination, it was observed that during intestinal peristalsis the distance between the peritoneum and the viscera adjacent to the ventral region variated, showing not to be a good evaluation tool to quantify peritoneal fluid. The association of videos findings, notes, and images obtained during ultrasonography examination was used for quantification.

**Figure 1:**
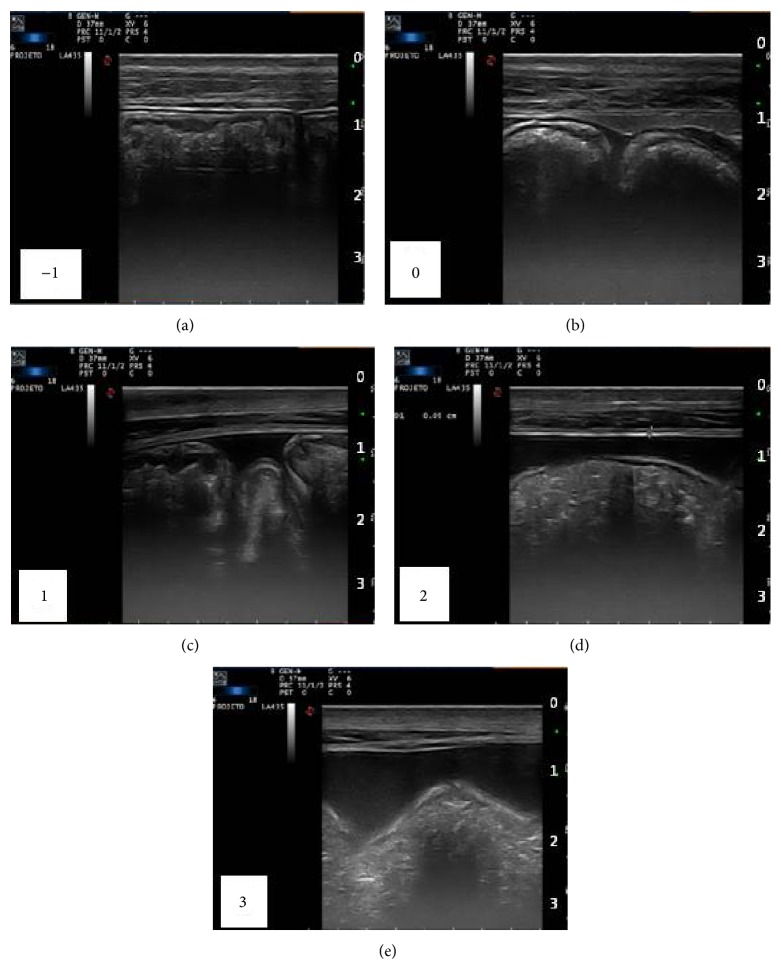
Longitudinal image of the ventral abdomen of horses from both groups, demonstrating the graduation scheme of free peritoneal fluid amount, being −1 (reduced), 0 (normal), 1 (slightly increased), 2 (moderately increased), and 3 (strongly increased).

The amount of free peritoneal fluid was standardized in a graduation system as follows: −1 (reduced), 0 (normal), 1 (slightly increased), 2 (moderately increased), and 3 (strongly increased), and normality was standardized as the more frequently image observed in animals prior to surgery (Figure 1).

**Figure 2:**
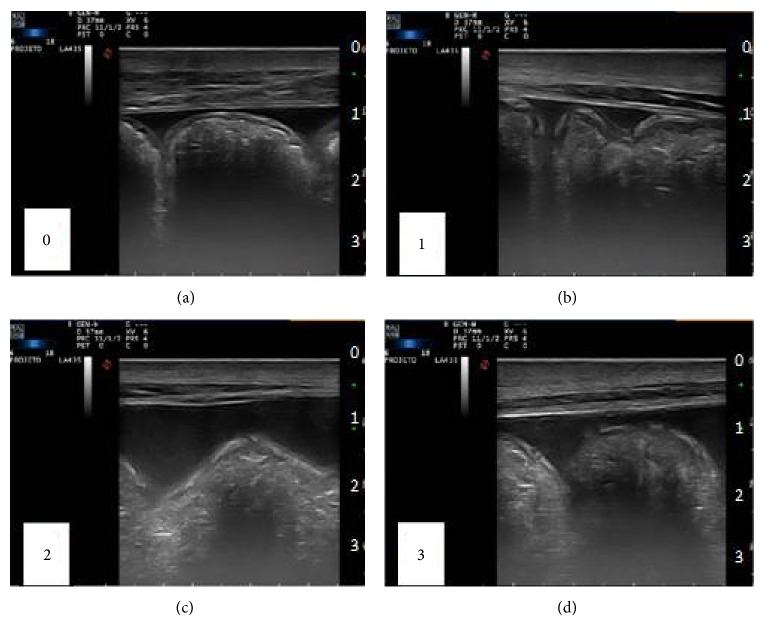
Longitudinal image of horses ventral abdomen from both groups. Demonstrative Scheme graduation of peritoneal fluid echogenicity, being 0 (normal), 1 (slight increase), 2 (moderate increase), and 3 (moderate increase associated with the presence of cellular debris).

Echogenicity was graded in a system of scores, as follows: 0 (normal), 1 (slightly increased), 2 (moderately increased), and 3 (moderately increased, associated with the presence of cellular debris), and normality was established as the appearance of liquid prior to surgical procedure (Figure 2).

The peritoneal fluid samples were collected at the most ventral region of the abdomen using a 40 × 12 mm hypodermic needle and tubes containing 3.8% sodium citrate at a 9 : 1 (liquid : anticoagulant) ratio (v/v) for D-dimer evaluation, EDTA tubes for cytology count, and tubes with no anticoagulant for physicochemical tests. The samples for the D-dimer were centrifuged for 15 minutes immediately after collection, using a 1000 G rotation. D-dimer analysis was performed using the latex agglutination test, according to the manufacturer's instructions.

Fifteen days after the initial surgery, a new surgical approach was performed. The animals received 700 g of magnesium suphate 36 hours before the procedure and were subjected to 24 hours fasting, in order to small colon emtying and easier handling of the segments. Prior to surgery, the left abdomen was shaved. Rectal palpation was proceeded in order to assess the anatomic topography at the site of cannulae insertion, avoiding iatrogenic punctures. The procedure was performed in standing position with identical sedation protocol to that used in laparotomy.

Laparoscopic equipment was composed of electronic insufflator CO_2_, Light Source Xenon 180 W, illumination cable, microcamera with processor, rigid endoscope (10 mm diameter, 57 cm length of 0 and 30 degrees) cannula EndoTIP TM (10 mm), laparoscopic and conventional surgical instruments, TV monitor, image recording system comprising a notebook, and a capture plate.

The surgical access area underwent local anesthesia with 2% lidocaine associated with vasoconstrictor. Antisepsis was performed; the skin incision was approximately 2 cm; the access was created through the introduction of video-assisted cannula (EndoTIP TM) with rigid endoscope inside. The pneumoperitoneum was induced. The abdominal pressure was maintained during the procedure between 12 and 15 mmHg, varying according to each animal supported volume. A systematic evaluation of abdominal cavity was established to perform a complete scan of structures amenable to evaluation through the left observation.

After abdominal cavity evaluation, two new anesthetic blocks points of approximately 1.5 cm where performed for cannulas insertion (11 mm); atraumatic intestinal graspers (Babcock) were introduced. A detailed evaluation of the small colon and enterotomy region was performed with Babcock forceps, supporting mesocolon dorsally; the optical was advanced to the right side of the abdomen in order to observe the laparotomy wound region. The images were stored for later evaluation.

The graspers were removed and the safety valves of the cannulas were opened allowing the release of the CO_2_; the cannulas were removed and finally withdrew the EndoTIP by performing rotational movements that promoted muscle layers approximation; the skin synthesis was performed in simple separated standard with nylon 0.

**Figure 3:**
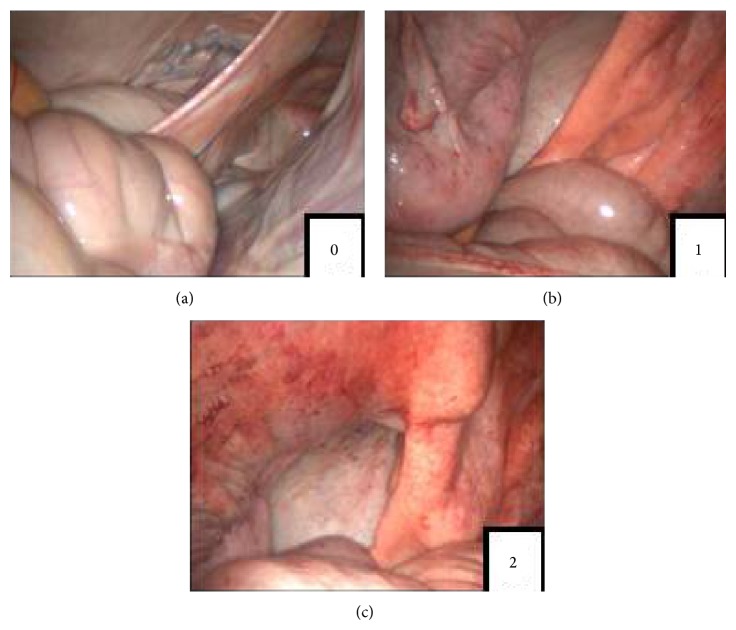
Score system of diffuse peritoneal reactivity, being 0 (no diffuse reactivity), 1 (mild diffuse reactivity), and 2 (moderate diffuse reactivity).

In order to facilitate the images evaluation scores of diffuse peritoneal reactivity were created, being 0 (no diffuse reactivity), 1 (mild diffuse reactivity), and 2 (moderately diffuse reactivity) (Figure 3).

The Wilcoxon test [25] was used to compare the median of each variable between the study groups. Later, comparisons between groups were performed at all times. Statistical analysis was performed with the PROC Npar1way [26] and statistical significance was defined as *P* < 0.05.

## 3. Results

The applied ultrasonographic methodology allowed the evaluation of small colon segments in approximately 85% of patients in different times, and possibly among these, only in a small proportion, the segment subjected to enterotomy was found, being rarely observed segments with wall thickening and architecture regularity loss.

Ventral abdomen ultrasonograph examination allowed the assessment of peritoneal fluid quantity, echogenicity, homogeneity, flocculation, and fibrin presence.

No difference between groups was observed for the amount of free peritoneal fluid; however an individual variation among animals was observed. Based on employed graduation scale it was found that animals maintained their individual volume regardless of the surgical procedure. After the 10th day after surgery there was a slight increase in the amount of free fluid in both groups, which is represented by the increase of one score in scale.

Relying on the proposed graduation scale, peritoneal echogenicity increased after 12 hours of surgery for both groups. Increased echogenicity persisted until the 14th day, however, with progressive reduction after the 6th day postoperatively. Although there was no significant difference between groups, the TG showed persistence of increased echogenicity in relation to the CG (Table 1).

Fibrin clots and irregularities in the parietal peritoneum were present between the 4th and 6th day in both groups.

After 12 hours of surgery, all peritoneal fluid samples presented red color; for TG the red color was maintained throughout the treatment period. Significant difference was obtained between groups (*P* < 0.0470), with 48% of red samples in CG and 72% for TG. In subsequent analysis, peritoneal fluid acquired gradually decreasing turbidity and coloration tends to be normal.

After laparotomy, there were an increased number of neutrophils, mononuclear cells, lymphocytes, and macrophages in both groups peritoneal fluid; all parameters increased in relation to the normal range [27]; however, no significant difference was observed between the groups.

For peritoneal RBC a significant difference between the groups was observed (*P* < 0.0365) when all times were combined (median value: CG-33900 and TG-140700 RBCs/*μ*L) and at M3 (*P* < 0.0358) and M4 (*P* < 0.0122) (Table 2).

The baseline values of D-dimer were below the reference values (4.0 to 88.0 ng/mL) [22] with detected concentrations <0.5 ng/mL. After surgery, considerably higher values were obtained for the CG, with median values of 4000 ng/mL (CG) and 1500 ng/mL (TG). However, there were no significant differences (*P* = 0.0745) between groups when all time-points were combined or not (Table 3).

No changes in visceral topography were observed on laparoscopic exam. The quantity and quality of peritoneal fluid evaluated macroscopically were compatible with ultrasonographic findings and did not differ between groups.

During small colon manipulation with atraumatic forceps, it was observed that adjacent regions to enterotomy had become covered with a thin layer of fibrinous exudate and presented reactive and friable to manipulation, rapidly becoming bleeding in both groups, but with greater intensity in TG.

Although there was no significant difference in diffuse peritoneal reactivity between groups, there was evidently greater reactivity in TG. CG showed 60% of score 0, 20% of score 1, and 20% of score 2 and TG showed 60% of score 1 and 40% of score 2.

**Figure 4:**
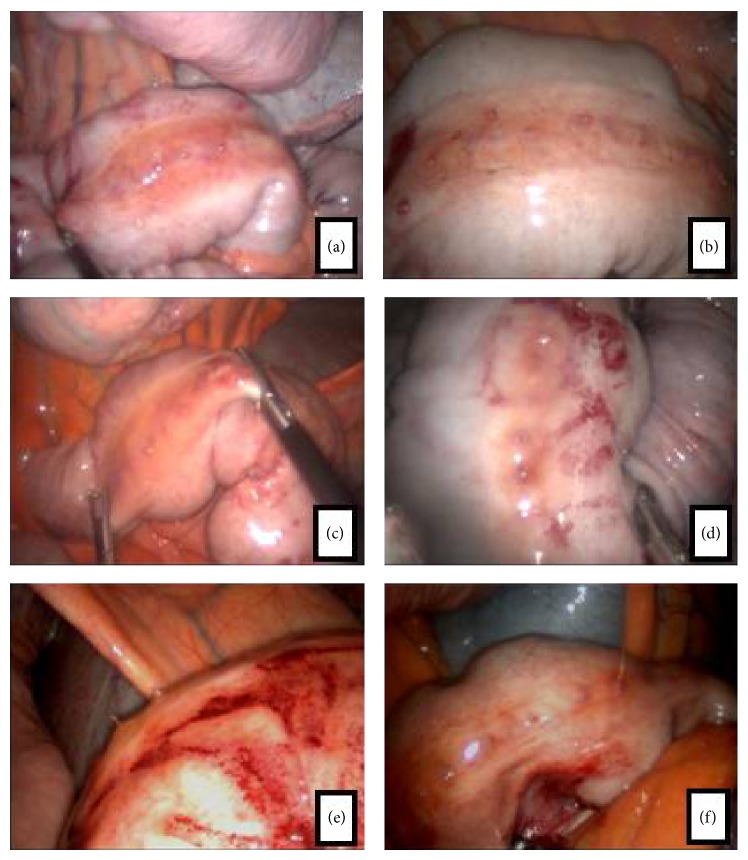
Laparoscopic image of small colon region subjected to enterotomy. (a), (b), and (c) control group (small colon enterotomy region covered by fibrin); (d), (e), and (f) treated group (small colon enterotomy region covered with fibrin; segments presenting hemorrhagic soon after manipulation).

Invariably, the region of the enterotomy to both groups was covered by fibrin. On the 15th day after enterotomy the suture performed with catgut was preserved. No difference between groups was observed for fibrin deposition over enterotomy region (Figure 4).

## 4. Discussion

Moore and Hinchcliff [12] described that an effective heparin dosage will be around 150 UI/Kg every 12 hours and suggest that a decreasing regimen should be used to avoid side effects, as anemia, hemorrhage, thrombocytopenia, and painful swelling at injection sites. In spite of no reduction on dosage during our treatment we observed no hemorrhage and a transient anemia and thrombocytopenia, which were rapidly reversible after discontinuation of treatment. Heparin probably results in microcirculation accumulation of RBC and platelets, which explains the rapid recovery of such amounts [12, 28–30].

Although transrectal ultrasonography is the most appropriate method for evaluating small colon [16], transabdominal route was chosen to avoid interference on enterotomy healing. The applied methodology did not allow the access to the small colon in all the exams. Small colon has been accessed in approximately 85% of patients in different times. Furthermore, among these 85%, only in a small proportion changes in the evaluated segments were observed; probably the applied methodology has not been adequate to evaluate the specific segment subjected to enterotomy.

Increased echogenicity observed 12 hours after surgery in both groups was assigned to cellular migration in response to abdominal inflammatory process and intracavitary surgical bleeding. The difference observed between groups for echogenicity possibly occurred due to heparin treatment, which resulted in maintenance of the displacement of RBCs for peritoneal fluid, since the high erythrocyte count, macroscopic aspect, and a gradual decreased in echogenicity for TG after the suspension of treatment.

Despite the fact that the difference in echogenicity did not show statistical significance between groups, it was observed that the TG showed higher echogenicity. Probably a study with a large number of animals should demonstrate statistical difference.

Postoperative peritonitis, fibrin presence, and peritoneal irregularities observed during this study corroborate with literature information that reports increased echogenicity due to high cellularity, loss of homogeneity, fibrin deposition, and presence of cellular debris [16, 31].

Although TG had presented higher peritoneal fluid echogenicity, it cannot be inferred by ultrasonographic examination that these animals had higher peritoneal reactivity, since the high liquid cellularity corresponded mainly to erythrocytes, with no difference between groups for the presence of fibrin, cellular debris, and thickening of the peritoneum and intestinal walls.

Ultrasonography allowed peritoneal cavity inflammation and bleeding due to heparin treatment identification but did not allow detailed tracing of small colon. Transabdominal ultrasound use may have influenced the sensitivity to detect small colon changes.

Although there was no significant difference in the D-dimer concentration between groups, higher values tended to be observed for animals in the CG, which is consistent with the notion that abdominal surgery results in a state of hyperfibrogenesis and hyperfibrinolysis [22]. Because the D-dimer fragment is released exclusively during fibrin lysis by plasmin, it was inferred that the animals in the heparin-treated group, despite undergoing the same surgical procedure, had lower D-dimer concentrations due to reduced formation and deposition of fibrin and consequently lower cleavage rates of fibrin.

D-dimer values are directly related to poor prognosis and high mortality; the indication for the use of heparin in hypercoagulable states and after abdominal surgery in equines was strengthened by the results of our study.

Although the animals in the present study did not show abdominal adhesions, our results highlight the potential use of heparin for prophylaxis in cases of abdominal adhesions. However, further clinical studies should evaluate the use of heparin in equines suffering from colic syndrome and those subjected to laparotomy.

Laparoscopy was performed through the left abdominal approach, a procedure which according to Galuppo et al. [17] allows small colon visualization. No visceral topography changes were observed in evaluated animals, being the structures localized as described by Galuppo et al. [17] and Silva et al. [32]. It was observed that both groups had mild diffuse peritoneal reactivity; however TG showed higher reactivity. Moreover, these animals responded to visceral manipulation with more frequent formation of hemorrhagic areas at the contact points with the graspers. It was expected that the TG presented less fibrin deposition over enterotomy region; however no gross difference between groups was observed.

Laparoscopic examination allowed a detailed abdominal cavity and enterotomy area analysis, demonstrating no significant difference on fibrin deposition over enterotomy region; however without statistical significance, more diffuse peritoneal reactivity to visceral manipulation on TG was observed.

The association of peritoneal fluid evaluation, laparoscopy, and ultrasonography allowed detailed access to the abdominal cavity. Ultrasonography and peritoneal fluid evaluation allowed the assessment of diffuse peritoneal inflammation, while laparoscopy allowed the evaluation of peritoneal inflammation and a detailed analysis of the segment subjected to enterotomy.

No gross beneficial reactions resulting from the use of heparin on peritoneal reactivity were observed; however, it was observed by D-dimer evaluation that the TG had less fibrin deposition, which is directly related to a lower rate of abdominal adhesions formation.

## Figures and Tables

**Table 1:** Peritoneal fluid echogenicity evolution on ultrasonograph using the employed score system (animals percentage).

Moment	Group	Score 0	Score 1	Score 2	Score 3
M0	CG	100%			
	TG	100%			

M1	CG			80%	20%
	TG			80%	20%

M2	CG			100%	
	TG			80%	20%

M3	CG			100%	
	TG			100%	

M4	CG		60%	40%	
	TG			100%	

M5	CG	20%	60%	20%	
	TG		40%	60%	

M6	CG	60%	20%	20%	
	TG		100%		

M7	CG	80%	20%		
	TG		100%		

No significant difference between groups. 0: normal, 1: mild increase in echogenicity, 2: moderate increase in echogenicity, and 3: moderate increase in echogenicity accompanied by the presence of fibrin and cellular debris.

**Table 2:** Comparison of the total peritoneal RBC count between the control (GC) group and the treated group (TG) at different times (RBCs/*μ*L).

Moment	CG			TG		
	Median	Q1	Q3	Median	Q1	Q3
M0	6350	5550	9300	1650	340	4750
M1	390000	218400	475000	1306500	301150	2532600
M2	681050	226125	1315550	2632600	1240675	3085350
M3	**88000** ^*^	63800	160800	**572850** ^*^	462300	1708500
M4	**30150** ^*^	8800	39600	**321600** ^*^	261300	522600
M5	8800	5500	12600	83125	25550	140700
M6	7850	6050	10950	48825	15800	60300
M7	33900	12700	70350	9550	4200	44500

^*^Significant difference between groups (*P* < 0.05). Q1: first quartile; Q3: third quartile.

**Table 3:** Comparison of the peritoneal D-dimer concentrations between the CG and TG at different time-points.

Time-point	D-dimer (ng/mL)					
	Control group			Treated group		
	Median	Q1	Q3	Median	Q1	Q3
M0	0	0	0	0	0	0
M1	32000	1000	32000	8000	8000	16000
M2	8000	8000	8000	4000	2000	32000
M3	8000	4000	16000	2000	1000	8000
M4	4000	4000	8000	2000	2000	8000
M5	2000	1000	4000	500	0	1000
M6	4000	500	4000	500	500	1000
M7	500	500	2000	0	0	2000

There was no significant difference between groups. Q1: first quartile; Q3: third quartile.
